# Selection and Validation of Reference Genes in *Dendrocalamus brandisii* for Quantitative Real-Time PCR

**DOI:** 10.3390/plants13172363

**Published:** 2024-08-24

**Authors:** Jutang Jiang, Changhong Mu, Yucong Bai, Wenlong Cheng, Ruiman Geng, Junlei Xu, Yuping Dou, Zhanchao Cheng, Jian Gao

**Affiliations:** Key Laboratory of National Forestry and Grassland Administration, Beijing for Bamboo & Rattan Science and Technology, International Center for Bamboo and Rattan, Beijing 100102, China; jiangjutangtea@163.com (J.J.); muchanghong@icbr.ac.cn (C.M.); bai.yucong@icbr.ac.cn (Y.B.); chengwenlong@icbr.ac.cn (W.C.); gengruiman@icbr.ac.cn (R.G.); xjl@icbr.ac.cn (J.X.); douyuping@icbr.ac.cn (Y.D.)

**Keywords:** *Dendrocalamus brandisii*, reference genes, qRT-PCR, normalization, *EF-1-α-2*

## Abstract

*Dendrocalamus brandisii* (Munro) Kurz is a sympodial bamboo species with a wide distribution in tropical and subtropical regions. Due to its remarkable regenerative ability and exceptional flavor, this species plays a pivotal role in bolstering the economies of numerous nations across these regions. We recently published a high-quality genome of this species. To date, no study results have identified the optimal reference genes for quantitative real-time polymerase chain reaction (qRT-PCR) normalization in *Dendrocalamus brandisii*. qRT-PCR offers a highly accurate and effective approach to analyzing gene expression. However, the precision of the resulting quantitative data hinges on the correct choice of reference genes. Twenty-one potential reference genes were identified from the *D. brandisii* transcriptomes. Their expression in 23 samples, including 8 different tissue organs and 15 samples of *D. brandisii* under various treatment conditions, were evaluated through qRT-PCR. Subsequently, four software programs—Delta CT, geNorm, NormFinder, and RefFinder—were employed to compare their expression stability. The results revealed that the selection of optimal reference genes varied based on the particular organ and condition being examined. *EF-1-α-2* consistently exhibits the most stable expression across diverse tissues, while *ACTIN-1*, *TUBULIN-1*, and *EF-1-α-2* were the most consistent reference genes in roots, culms, and leaves under various treatments, respectively. In this study, we identified and characterized appropriate internal genes utilized for calibrating qRT-PCR analyses of *D. brandisii* across different tissue organs and under various treatments. This research will provide key insights for advancing the study of gene functionality and molecular biology in *D. brandisii* and related species

## 1. Introduction

*Dendrocalamus brandisii* (Munro) Kurz is a prominent tropical and subtropical evergreen sympodial bamboo species. It belongs to the *Dendrocalamus* genus within the Bambusoideae subfamily of the Gramineae family. This species originates from the southern and northeastern regions of India, as well as Myanmar. Its primary distribution encompasses southern China, extending to various South and Southeast Asian nations, and even as far as Ethiopia, Bosnia, Herzegovina, and Brazil [1,2]. *D. brandisii*, in conjunction with *D. hamiltonii* and *D. aspera*, is heralded as one of the three paramount sweet bamboo species not only in China, South Asia, and Southeast Asia but globally [3]. The shoots of this bamboo stand out for their unparalleled quality, characterized by a distinct sweetness and the highest concentrations of glutamate and sugar among bamboo species harvested for shoots in China. Consequently, *D. brandisii* has established itself as a leading source of premium edible bamboo shoots, catering to both local consumption and international export requirements [4].

qRT-PCR is a technique employed in molecular studies to measure nucleic acid levels and analyze gene expression [5]. Due to its pronounced precision and sensitivity, qRT-PCR has been increasingly adopted across various sectors of biological research [6,7,8]. A consistent reference gene is vital for accurately assessing the relative expression of target genes. However, to ensure accurate and reliable outcomes, it is imperative to employ a reference gene to standardize gene expression. This normalization mitigates potential discrepancies stemming from variations in experimental procedures, such as sample volume, RNA integrity and amount, the efficiency of enzymatic reactions, and the performance of PCR [9,10]. When utilizing qRT-PCR to compute the relative expression patterns of target genes, it is vital to include reference genes that demonstrate consistent stability for precise adjustment and standardization, thereby enhancing the precision of quantitative outcomes [11,12]. The reference genes frequently employed are predominantly housekeeping genes such as actin (*ACT*), tubulin (*TUB*), elongation factor 1-α (*EF-1-α*), glyceraldehyde-3-phosphate dehydrogenase (*GAPDH*) and ribosomal RNAs (*18S rRNA* or *28S rRNA*). Nonetheless, some datasets have pointed out that the expression patterns of these maintenance genes can significantly change across different experimental treatments [13,14,15]. Advancements in molecular biology technologies, including Affymetrix GeneChip, microarrays, and high-throughput sequencing, have led to the discovery of numerous novel reference genes in model plants such as *Arabidopsis thaliana* [16], *Populus trichocarpa* [17], and *Oryza sativa* [18]. Additionally, in plants that are not model organisms, the absence of genetic and genomic sequence data poses challenges. The chosen reference genes are typically ascertained based on the orthologous sequences of the commonly reported reference genes in standard plant models [19]. Hence, it is crucial to judiciously choose suitable reference genes based on the specific experimental settings [20]. Additionally, statistical analysis tools like geNorm, Delta CT, NormFinder, and RefFinder are commonly employed as robust tools for evaluating gene expression stability in qRT-PCR normalization [21,22,23]. Validation of reference genes has been carried out across a myriad of plant species, such as *Euscaphis* [24], *Rubus* [25], pitaya [15], *Phyllostachys edulis* [26], *Jatropha curcas* [27], *Isatis indigotica* [28], *Achyranthes bidentata* [29], *Kentucky bluegrass* [30], *Salix matsudana* [31], *Rhododendron* [32], and *Dendrobium officinale* [33].

Based on the transcriptomic sequencing data of *D. brandisii*, CDS sequences retrieved from the genome, and other information, 21 genes were identified as prospective candidates for reference gene selection. We assessed their expression consistency across different experimental conditions using qRT-PCR. This encompassed eight tissues (roots, culms, leaves, buds, shoots, shoot sheaths, root primordium, and branches) and roots, culms, and leaves under drought stress (PEG) [34] and hormonal treatments (SA, ETH, MeJA, ABA) [35] in order to identify reference genes suitable for normalization across different experimental conditions. Subsequently, we performed an in-depth evaluation of the expression consistency of the candidate genes using analytical tools like geNorm, NormFinder, and Delta CT. Additionally, to corroborate our results, we normalized the expression patterns of *DhA16G008210* and *DhA12G022730* across 15 distinct tissues using the most consistent genes derived from our transcriptomic dataset.

## 2. Results

### 2.1. Selection and Amplification Efficiency of Reference Genes

Based on the selection criteria derived from the transcriptome sequencing data, we chose the two genes with the least expression variation from 12 traditional reference gene families (*18S rRNA*, *ACTIN*, *CYP*, *EF-1-α*, *GAPDH*, *NTB*, *RPL*, *TEF*, *TUBULIN*, *NAC*, *UB2C*, *UBC*). After screening, a total of 21 genes (*18S-1*, *18S-2*, *ACTIN-1*, *ACTIN-2*, *CYP-1*, *CYP-2*, *EF-1-α-1*, *EF-1-α-2*, *GAPDH-1*, *GAPDH-2*, *NAC-2*, *NTB-2*, *RPL-2*, *TEF-1*, *TEF-2*, *TUBULIN-1*, *TUBULIN-2*, *UB2C-1*, *UB2C-2*, *UBC-1*, *UBC-2*) were chosen as potential reference genes from our literature search. The specifics of gene shorthand, primer sequences, product size, correlation coefficient (R^2^), and amplification efficiency are shown in Table 1. The leaves, culms, sheaths, branches, roots, shoots, bud primordium, bamboo buds at the base of the culms, and the different treatment roots, culms, and leaves served as specimens for the selection of potential reference genes. The specificity of the primer sets was assessed using the qRT-PCR melt curve. All primers for the target candidate reference genes exhibited a distinct single amplification peak (Figure S1). In other words, all primers for the reference genes demonstrated specificity and were suitable for qRT-PCR-based gene expression analysis. The amplification efficiency (E) of all 21 reference gene reactions varied from 92.9% for *ACTIN-2* to 187.0% for *TUBULIN-2*, and the correlation coefficient (R^2^) ranged from 0.9347 to 0.9888 (Table 1).

### 2.2. Ct Value of 21 Reference Genes

**Figure 1:**
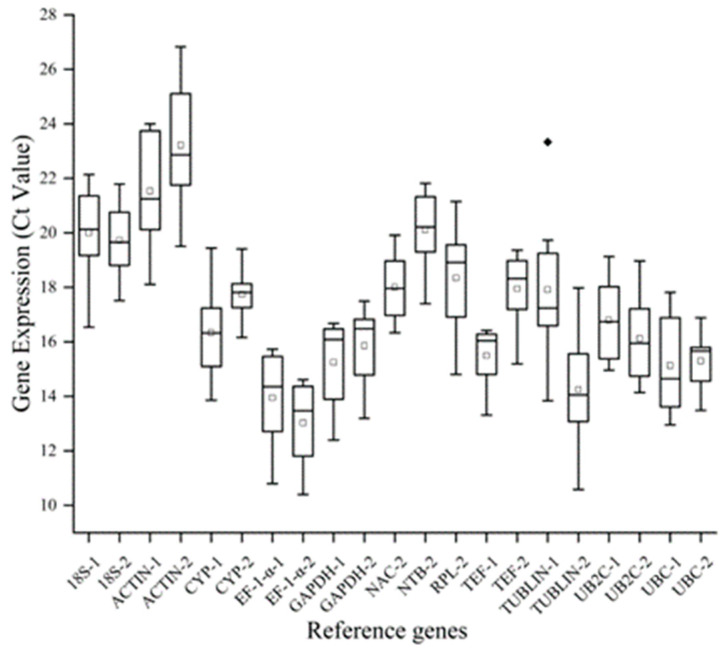
Ct values of the 21 candidate reference genes. The line across the box represents the median. The boxes represent the 25/75 percentiles. The lower and upper dashes depict the minimum and maximum value. White circles represent the mean values, while black diamond indicate outliers.

To evaluate the expression consistency of the 21 potential reference genes across various tissues and under different treatment conditions, the transcriptional abundances were represented as Ct values. The Ct values for all 21 potential reference genes are depicted in Figure 1. Across all samples, Ct values varied between 10.394 (*EF-1-α-2*) and 26.826 (*ACTIN-2*), while the mean Ct values ranged from 13.036 (*EF-1-α-2*) to 23.219 (*ACTIN-2*). Since Ct values are inversely proportional to gene expression levels, *EF-1-α-2* exhibited high expression while *ACTIN-2* showed low expression levels.

### 2.3. Stability of Gene Expression for Reference Candidates Using Three Different Algorithms

The consistency in expression for all reference genes was analyzed using NormFind-er, geNorm, Delta CT, and RefFinder. The templates were divided into the following four different experiment groups: (1) eight tissues (leaves, culms, sheaths, branches, roots, shoots, bud primor-dium, bamboo buds at the base of culms), (2) leaves under five different treatments, (3) roots under five different treatments, and (4) culms under five different treatments.

#### 2.3.1. geNorm Analysis

The analysis results are as follows: The geNorm software (v 3.5) assessed the stability of reference genes by computing the mean expression stability measurement (M) values. A smaller M value signifies greater stability in gene expression. Additionally, a gene possessing an M value below 1.5 is considered suitable for use as a reference gene. For the tissue group, *EF-1-α-2* and *EF-1-α-1* emerged as the most consistent genes, displaying the smallest M values, while *TUBULIN-1* had the highest M value. Within the leaf processing group, *EF-1-α-1* and *TEF-1* emerged as the most consistent reference genes, while *CYP-2* showed the greatest variability. Conversely, in the culm processing group, *EF-1-α-2* and *EF-1-α-1* were identified as the most consistent genes, with *UBC-1* being the least consistent. Finally, within the root processing group, *ACTIN-1* and *GAPDH-1* stood out as the most consistent reference genes, whereas *NTB-2* showed the most fluctuation (Table 2).

#### 2.3.2. NormFinder Analysis

The NormFinder software (v 0.953) determined the consistency of the reference genes using the stability (S) value. The stability values assessed by NormFinder are presented in Table 3. Within the tissue group, *TEF-2* emerged as the most stable gene, while *TUBULIN-1* was identified as the least stable, similar to the results obtained from geNorm analysis. Within the processed leaves group, *EF-1-α-2* emerged as the most consistent gene, with *CYP-2* being the least consistent. For the processed culms group, *TEF-1* stood out as the most stable and *UBC-1* was identified as the least stable. In the context of the processed roots group, *TEF-1* was the most reliable gene, with *NTB-2* showing the least stability.

#### 2.3.3. Delta CT Analysis

In the Delta CT software (v 4.1.3), the consistency of each reference gene’s expression is gauged by the mean standard deviation (mSD) value. A lower mSD value indicates greater stability. Table 4 presents the stability values as determined by the Delta CT method. For the tissue, processed leaves, and roots groups, *EF-1-α-2* emerged as the most stable gene. Meanwhile, in the processed culms group, *TUBULIN-1* was identified as the most stable gene.

#### 2.3.4. Comprehensive Ranking Analysis

Given the distinct algorithms employed by the three analytical software applications (geNorm, NormFinder, and Delta CT), there is not a complete consensus on the most consistent reference genes. To ensure the most reliable and precise outcomes, it is imperative to consider the results from all three software tools. Thus, the stability rankings from these three tools were integrated into RefFinder to provide a holistic assessment of gene expression stability for this study. A lower sequence value indicates greater gene expression stability. The findings indicated that the *EF-1-α-2* gene exhibited the highest stability as a reference gene within the tissue group and processed leaves group. *TUBULIN-1* and *ACTIN-1* emerged as the most consistent gene in the processed culms group and processed roots group, respectively (Table 5).

### 2.4. Validation of Candidate Reference Genes

**Figure 2:**
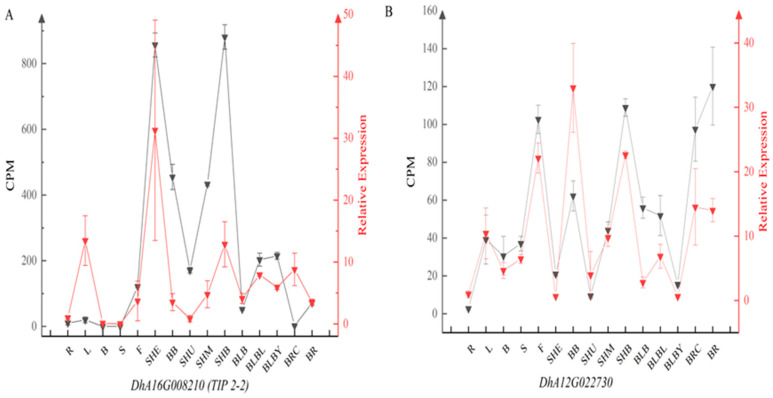
Relative expression of *DhA16G008210* (*TIP 2-2*) (**A**) and *DhA12G022730* (**B**) in *D. brandisii*. CPM: counts per million. The abbreviations on the X-axis represent different tissue types from the published *D. brandisii* transcriptome. R: root base, L: leaf, B: branch, S: culm, F: flower, SHE: sheath, BB: buds at the base of the culm, SHU/SHM/SHB: the upper, middle, and base of 50 cm tall shoots, BLB: bud primordium, BLBL: current-year bud, BLBY: annual bud, BRC: root primordium, BR: root tip.

Based on the transcriptomic data of different *D. brandisii* tissues, we found that *DhA16G008210* (*TIP 2-2*) and *DhA12G022730* were significantly differentially expressed among the different tissues. To validate the robustness of the chosen stable reference genes, we assessed the relative expression patterns of *DhA16G008210* and *DhA12G022730* using the top-performing reference genes across various *D. brandisii* tissues. The reference gene *EF-1-α-2*, identified as the most stable from the aforementioned analyses, was employed for qRT-PCR evaluations and then compared with the transcriptome data. When the most stable gene of *EF-1-α-2* was used for normalization in different tissues, the relative expression trends in *DhA16G008210* and *DhA12G022730* were similar to the transcriptome data (Figure 2). These findings suggested that the chosen reference gene exhibited consistent stability across the tissues.

## 3. Discussion

qRT-PCR is an important method for determining gene transcript levels and understanding the biological functions of target genes in plants [36]. Given that the precision and dependability of qRT-PCR outcomes can be influenced by tissue variations, sample uniqueness, RNA quality, and other factors, a consistent reference gene is essential for proper data normalization [37]. Ideally, reference genes should exhibit consistent expression across various developmental phases, tissues, or organs, and under diverse experimental conditions, with an expression pattern mirroring that of the target gene [38,39]. Traditionally, certain housekeeping genes like *ACTIN*, *TUB* and *18S* (cytoskeleton structure), *GAPDH* (metabolic processes), and *TEF* and *EF-1-α* (protein synthesis) are frequently used as internal reference genes. In prior research, the expression stability of reference genes demonstrated considerable variation across distinct species, genetic variants, developmental stages, tissue types, and experimental conditions. In raspberry and blackberry, the authors found that *EF-1-α* and *18S* were identified as the primary reference genes. However, several research findings suggest that *18S* may not be a suitable reference gene for species like *Stevia rebaudiana* and *Zanthoxylum bungeanum*, further underscoring the species-specific nature of suitable reference genes [25]. In radish, *TEF2* and *RPII* exhibited robust expression stability across a diverse array of tissue types. However, in Duan’s research, in terms of different organs, various developmental stages of pistils, and across the entirety of sampled radish tissues, *UPR*, *UP2* and *GAPDH*, respectively, were pinpointed as the best reference genes [40]. Conversely, *RPII* emerged as the most dependable reference gene solely in the context of biotic and abiotic stress conditions. Therefore, it is crucial to rigorously select appropriate reference genes for normalization, tailored to the specific species and experimental conditions of the study.

In order to circumvent the limitations of relying solely on a single software analysis, we employed three bioinformatics tools (geNorm, Delta CT, NormFinder) to assess the consistency of expression for potential reference genes. In geNorm, the core approach for determining gene stability involves employing the 2^−ΔCt^ value for each gene to compute the M value [21]. The NormFinder algorithm functions similarly to geNorm, employing the 2^−ΔCt^ value as a measure of relative expression to determine the stability of gene expression. A significant advantage of NormFinder over geNorm lies in its capacity to accommodate sample categorization, a critical element impacting gene expression stability [22]. Delta CT is calculated by deducting the CT value of a consistent reference gene from the CT value of the gene being studied. This approach is highly favored for its ease of use and effectiveness, especially when it comes to comparing the expression of a particular gene among various samples or under different experimental conditions [23]. The rankings from different programs exhibited some notable discrepancies. For example, in various tissue organs, geNorm and Delta CT identified *EF-1-α-2* as the best reference gene (Table 2 and Table 4), while NormFinder assessed *TEF-2* as the optimal choice (Table 3). Ranking inconsistencies across these tools, as observed in related research [41,42], are likely attributed to the distinct computational algorithms that each software employs [43]. Combining results from several analytical tools can reduce inaccuracies when assessing the stability of potential reference genes. Here, we employed RefFinder to provide a comprehensive assessment [44,45,46,47]. By consolidating the findings from the three analytical tools, we produced a consolidated ranking to identify the most consistent reference gene. Upon comprehensive analysis of the 21 candidate genes, we identified genes *EF-1-α-2*, *ACTIN-1*, *TUBULIN-1*, and *EF-1-α-2* as the ideal reference gene for assessing gene expression across various tissues and under different treatments in roots, culms, and leaves, respectively.

To confirm the consistency of our proposed reference genes, we randomly selected two genes (*DhA16G008210* (*TIP 2-2*) and *DhA12G022730*) from the transcriptome data of 15 tissue organs and assessed their relative expression levels across these 15 organs using qRT-PCR. When employing *EF-1-α-2* for normalization, the expression patterns of these two genes closely aligned with the expression patterns observed in the transcriptome data. These findings highlight that *EF-1-α-2* is the most reliable reference gene for studying the expression of pivotal genes across various tissue organs in *D. brandisii*.

To the best of our knowledge, this is the first comprehensive evaluation of the stability of potential reference gene expression in qRT-PCR studies involving *D. brandisii.* In this study, we selected 21 traditional housekeeping genes to identify the most consistent ones for qRT-PCR standardization. Based on calculations from geNorm, Delta CT, NormFinder and RefFinder programs, *EF-1-α-2*, *ACTIN-1*, *TUBULIN-1*, *EF-1-α-2* were recognized as the ideal reference genes across different tissues and treatments in roots, culms, and leaves. Additionally, we analyzed the expression of *DhA16G008210* (*TIP 2-2*) and *DhA12G022730*, with *EF-1-α-2* being recognized as the prime reference gene for qRT-PCR standardization. The reference genes pinpointed in this research for various tissues and treatment conditions will serve as powerful tools during molecular biology research on *D. brandisii*.

## 4. Materials and Methods

### 4.1. Plant Materials

The wild bamboo materials were exclusively collected from Pu’er City, Yunnan Province (100.94370 E, 22.72578 N), a natural habitat of *D. brandisii*, including roots, culms, leaves, buds, shoots, shoot sheaths, root primordium and branches [48,49]. The treated bamboo samples were sourced from the International Center for Bamboo and Rattan. One-month-old seedings were treated with SA (200 μmol/L), ETH (1 mmol/L), MeJA (100 mmol/L), ABA (100 μmol/L), and 50%PEG to simulate drought and various hormonal treatments, aiming to select standardized reference genes under different conditions; after 7 days, samples from the roots, culms, and leaves were taken, cleaned, and surface-dried. For each biological replicate, we combined six plant samples, and the study incorporated a total of three such replicates. All specimens were instantaneously flash-frozen using liquid nitrogen, then conveyed under dry ice conditions and preserved at −80 °C.

### 4.2. Candidate Genes Selection and Primer Design

Drawing from the transcriptomic data previously sequenced in our laboratory, we chose 21 reference genes exhibiting consistent expression for normalization and to perform qRT-PCR validation assays, taking into account their CPM and fold change metrics. The sequence information for these genes can be found in Table S1. We used Primer Premier 5.0 to design the forward and reverse primers for these candidate reference genes, adhering to the following given criteria: melting temperatures (Tm) between 55 and 65 °C, GC percentage between 45 and 60%, primer sequences spanning 18–22 base pairs, with amplicon sizes between 150 and 250 base pairs. All primer sets were procured from Suzhou Genewise Technology Co., Ltd. (Suzhou, China). Primer specifics can be found in Table 1.

### 4.3. RNA Isolation and cDNA Preparation

The total RNA was isolated from different tissue and organs of *D. brandisii* using the Trizol Reagent (LABLEAD, Beijing, China) following the manufacturer’s protocol [50]. The RNA integrity was evaluated through 1.0% agarose gel electrophoresis, while its concentration and purity were ascertained using a NanoDrop2000 spectrophotometer (Thermo, Waltham, MA, USA). Only RNA samples exhibiting an A260/A280 ratio in the range of 1.8 to 2.2, and an A260/A230 ratio above 1.8 were selected for subsequent experiments. A PrimeScript RT kit was employed to convert the total RNA from each sample into cDNA (LABLEAD).

Each RNA sample underwent triplicate analysis, accounting for both biological and technical repetitions. A series of fivefold dilutions of the cDNA template were prepared to establish the standard curve’s slope, which in turn allowed for the determination of the amplification efficiency and correlation coefficient for every candidate gene.

### 4.4. qRT-PCR Analyses

qRT-PCR was executed on the LightCycler 480 PCR platform (Roche Applied Science, Basel, Switzerland) using 96-well plates and the SYBR Premix Ex Taq TM II reagent kit (LABLEAD). The amplification protocol started with an initial denaturation at 95 °C for 10 s, followed by 45 cycles of 95 °C for 5 s and 60 °C for 30 s. The melt curve analysis was conducted at the end of the amplification process, where the temperature was gradually increased from 65 °C to 90 °C to record the melt curves. Each 20 µL reaction mixture consisted of 10 µL TB Green Premix, 0.8 µL of each 10 mM primer, 2 µL of cDNA template, with the remaining volume adjusted to 20 µL with sterile water. To eliminate any technical variations, the entire set of samples for each gene was run on the same plate during each replication. The amplification efficiency of all primer pairs was assessed using fivefold serial dilutions of pooled cDNA (1/5, 1/25, 1/125, 1/625, and 1/3125).

### 4.5. Data Analysis

The average Ct value was used to gauge the expression magnitude of each potential reference gene. To holistically rank these candidates, their expression consistency was evaluated using the Delta CT method, along with the geNorm, NormFinder, and RefFinder software (https://blooge.cn/RefFinder/) tools. All steps were meticulously executed in line with the recommendations provided by the respective software.

### 4.6. Validation of Reference Genes

To corroborate the dependability of the chosen reference genes, we examined the expression patterns of the *DhA16G008210* and *DhA12G022730* genes across different tissues and growth phases of sweet bamboo, employing the most consistent reference genes pinpointed by RefFinder. The qRT-PCR procedure adhered to the aforementioned protocol, and the relative expression was determined using the 2^−ΔΔct^ approach [51]. Data collected from three biological replicates were analyzed using ANOVA and subsequently subjected to a Student’s *t*-test with the significance threshold set at *p* < 0.05.

## Figures and Tables

**Table 1:** Primers used for qRT-PCR normalization.

Genes	Forward Primer	Reverse Primer	Length (bp)	Efficiency (%)	Correlation Coefficient (R^2^)
*18SrRNA1*	AATGGGTGGGGAAAGATGT	TGGCTCAGTTGGAAGCTCT	216	114.7	0.9903
*18SrRNA2*	AGAAATGGGTGGGAAAAGA	TGGCTCAGTTGGAAGCTCT	219	110.5	0.9855
*ACTIN-1*	TGGCAGATTGATGCTAAGA	TCAGGTCGTGAACTGCAAG	192	106.0	0.9812
*ACTIN-2*	GGACGCAAGTGGAAACTTA	AAAAGGCCAAGCATAGCA	206	92.9	0.983
*CYP-1*	CAGTGATCAAAGGATGGATG	ACTGGGCGCTGAACTTCA	171	97.9	0.9793
*CYP-2*	ACTTCAAGCACAGCACACG	TCGATGGCCTGACAACAT	169	101.2	0.9765
*EF-1-α-1*	AAGCCTGGCATGGTTGTTAC	TCATCCTTGGAGTGGAAGC	179	111.4	0.9969
*EF-1-α-2*	GCTTCCAACTCCAAGGATGA	CCAGCTCAGCAAACTGACA	151	109.6	0.9904
*GAPDH-1*	GAATGCTAGCTGCACCACAA	AGCTTGCCATTCAAATCAGG	240	113.6	0.975
*GAPDH-2*	GAATGCTAGCTGCACCACAA	TGCTGCTGGGAATGATGTTA	188	118.5	0.9673
*NAC-2*	ATTTGCTCCCTGGGATCTTC	CTTTAGGTGCCTTGCCTTCA	227	110.4	0.9882
*NTB-2*	GCTTTGCATGGAAGGAACAT	GAAGCTCCAGGTTGTTGGAA	206	131.1	0.9370
*RPL-2*	CCAACAAGCTCTCCAGATCA	AAAGAGAGCTGGTGCTGGTT	184	128.0	0.9922
*TEF-1*	ACTGTCTTTTCCTGCCCATTC	CACACTCGTCAATCCATTCG	172	108.3	0.9857
*TEF-2*	TTCCTGGAGATGGACAAGGA	ACGTTGTTGTCTTGCAAATC	198	109.1	0.9886
*TUBULIN-1*	ACCATTGGAGGAGGTGATGA	AAGTTGTTGGCTGCATCCTC	188	121.3	0.971
*TUBULIN-2*	CGACCACAAGTTTGACCTCAT	CCTTGACAAAGCACCAGAT	189	187.0	0.9347
*UB2C-1*	TTGAAGGACCTGCAGAAGGA	TTCGGTGGCTTGAAAGGATA	170	120.2	0.9926
*UB2C-2*	TTGAAGGACCTGCAGAAGGA	TTCGGTGGCTTGAAAGGATA	195	128.2	0.9681
*UBC-1*	GGTGGCATTCAAGACAAAGG	ATGTGAGCAATCTCCGGAAC	180	119.2	0.9934
*UBC-2*	GCGATTTGTTTCTCGGATGT	ATTCCCGCTTGTTCTCACTG	203	109.4	0.9857

**Table 2:** Gene expression stability calculated by geNorm.

Genes	Tissues	Leaves	Culms	Roots
*18S-1*	0.798	0.764	0.541	0.897
*18S-2*	0.936	0.731	0.376	0.578
*ACTIN-1*	1.268	0.551	0.218	0.165
*ACTIN-2*	1.145	0.597	0.589	0.86
*CYP-1*	0.868	0.857	0.609	0.611
*CYP-2*	1.325	0.911	0.726	0.796
*EF-1-α-1*	0.322	0.186	0.145	0.264
*EF-1-α-2*	0.322	0.227	0.145	0.29
*GAPDH-1*	0.463	0.263	0.314	0.165
*GAPDH-2*	0.521	0.352	0.277	0.213
*NAC-2*	1.079	0.205	0.566	0.832
*NTB-2*	0.757	0.443	0.796	0.958
*RPL-2*	0.593	0.631	0.428	0.341
*TEF-1*	0.992	0.186	0.51	0.466
*TEF-2*	0.694	0.31	0.674	0.498
*TUBULIN-1*	1.597	0.801	0.235	0.419
*TUBULIN-2*	1.209	0.829	0.646	0.719
*UB2C-1*	1.405	0.388	0.699	0.675
*UB2C-2*	1.471	0.694	0.758	0.756
*UBC-1*	1.53	0.661	0.835	0.532
*UBC-2*	1.035	0.487	0.474	0.376

**Table 3:** Gene expression stability calculated by NormFinder.

Genes	Tissues	Leaves	Culms	Roots
*18Sr-1*	1.003	0.888	0.592	1.136
*18S-2*	1.21	0.791	0.325	0.774
*ACTIN-1*	1.754	0.583	0.36	0.341
*ACTIN-2*	1.264	0.59	0.58	0.987
*CYP-1*	1.057c	0.977	0.663	0.783
*CYP-2*	1.55	1.341	0.826	0.992
*EF-1-α-1*	0.651	0.158	0.361	0.34
*EF-1-α-2*	0.505	0.084	0.376	0.29
*GAPDH-1*	0.776	0.422	0.704	0.424
*GAPDH-2*	0.743	0.63	0.515	0.489
*NAC-2*	0.85	0.189	0.417	0.945
*NTB-2*	0.602	0.428	1.01	1.413
*RPL-2*	0.916	0.593	0.467	0.58
*TEF-1*	0.867	0.278	0.317	0.254
*TEF-2*	0.418	0.46	0.641	0.383
*TUBULIN-1*	1.994	0.972	0.326	0.415
*TUBULIN-2*	1.311	0.991	0.691	0.795
*UB2C-1*	1.642	0.646	0.739	0.793
*UB2C-2*	1.729	0.933	0.995	0.895
*UBC-1*	1.731	0.653	1.082	0.648
*UBC-2*	0.793	0.606	0.507	0.368

**Table 4:** Gene expression stability calculated by Delta CT.

Genes	Tissues	Leaves	Culms	Roots
*18S-1*	1.48	1.05	0.81	1.27
*18S-2*	1.6	0.98	0.69	0.99
*ACTIN-1*	1.99	0.86	0.67	0.76
*ACTIN-2*	1.7	0.87	0.84	1.18
*CYP-1*	1.51	1.15	0.87	1.01
*CYP-2*	1.89	1.42	0.98	1.17
*EF-1-α-1*	1.27	0.66	0.67	0.75
*EF-1-α-2*	1.21	0.65	0.68	0.73
*GAPDH-1*	1.32	0.71	0.88	0.78
*GAPDH-2*	1.31	0.86	0.76	0.82
*NAC-2*	1.45	0.68	0.75	1.15
*NTB-2*	1.3	0.79	1.14	1.54
*RPL-2*	1.42	0.88	0.74	0.87
*TEF-1*	1.44	0.78	0.69	0.75
*TEF-2*	1.24	0.78	0.86	0.8
*TUBULIN-1*	2.24	1.12	0.66	0.8
*TUBULIN-2*	1.73	1.14	0.87	1
*UB2C-1*	1.95	0.88	0.9	1.01
*UB2C-2*	2.02	1.08	1.1	1.07
*UBC-1*	2.05	0.93	1.21	0.91
*UBC-2*	1.42	0.89	0.77	0.77

**Table 5:** Gene expression stability calculated by RefFinder.

Genes	Tissues	Leaves	Culms	Roots
*18S-1*	10.38	9.66	11.72	12.45
*18S-2*	9.07	7.75	4.28	6.45
*ACTIN-1*	18.19	8.38	4.12	2.99
*ACTIN-2*	15.31	7.54	11.72	18.74
*CYP-1*	10.93	15.7	11.98	8.44
*CYP-2*	8.12	21	16.9	18.69
*EF-1-α-1*	3.66	2.74	3.41	3.94
*EF-1-α-2*	2.06	2.58	3.94	3.31
*GAPDH-1*	6.24	6.79	13.24	4.12
*GAPDH-2*	5.32	10.8	9.49	6.64
*NAC-2*	8.49	4.33	7.61	16.71
*NTB-2*	4.92	7.64	19.75	20.75
*RPL-2*	8.78	8.7	7.2	9.4
*TEF-1*	7.19	4	3.16	3.71
*TEF-2*	2.91	7.84	7.21	8.24
*TUBULIN-1*	21	13.07	3.13	8.9
*TUBULIN-2*	15.7	14.43	14.74	14.93
*UB2C-1*	16.13	12.41	11.02	14.48
*UB2C-2*	15.97	17.16	18.2	16.48
*UBC-1*	18.67	12.87	21	8.54
*UBC-2*	6.05	11.45	10.83	6.3

## Data Availability

The data and Supplementary Materials supporting the conclusions of this study are included within the article.

## Supplementary Materials

The following supporting information can be downloaded at: https://www.mdpi.com/article/10.3390/plants13172363/s1, Figure S1. Melt curve analyses of 21 reference genes; Table S1. Sequences of 21 reference genes.
