# Supplementary material for: Selection and Validation of Reference Genes in Dendrocalamus brandisii for Quantitative Real-Time PCR
Source: Plants (Basel). 2024 Aug 24;13(17):2363. doi: 10.3390/plants13172363 (PMC11396877; doi:10.3390/plants13172363)
Supplement: Supplementary file 1 [file plants-13-02363-s001.zip › Table S1. Sequences of 21 reference genes. .pdf]

18SrRNA-1

ATGGCGGGCGGCAAGATCCAGAAGAAGCGGCACGGCGGAGGCGCGGGAGGCGGCGG
CGGCCGGCTGCAGGGCGGGATCCCGTTCGAGAAGTCCAAGGGGCAGCACATCCTGCG
GAACCCAGCGCTGGTGGACTCCATCGTCGCCAAGGCCGGCCTCAAGCCCACCGACAC
CGTCCTCGAGATCGGGCCCGGCACGGGGAACCTCACTAAGCGCCTGCTCGAGGCCGG
CGTCAAGGCCGTCGTCGCCGTCGAGCTCGACCCGCGCATGGTGCTCGAGCTCAACCGC
CGTTTTCAGGGGCACCCCCTCTCCTCACGCCTCAAGGTTATCCAAGGAGATGTCCTTAA
ATGTGATCTCCCGTACTTTGATATCTGTGTGGCAAACATTCCATACCAGATTTCATCCCC
CCTTACATTCAAGCTTCTGTCACACCGTCCGATCTTTAGGTGTGCTGTGATCATGTTTCA
ACGTGAATTTGCCATGAGACTTGTAGCACAGCCTGGAGACAGTCTCTACTGCCGTCTC
TCAGTGAACGTGCAGCTCTTATCACGTGTGTCACATCTCCTGAAGGTTGGACGGAACA
ACTTCAGGCCTCCACCCAAGGTTGATTCATCAGTTGTGCGTATTGAGCCCAGGAAACC
CCTCCCTCCTGTTAGCTTCAAGGAGTGGGATGGACTTGTGAGGCTTTGTTTCAATCGAA
AGAACAAGACCTTGGGCTCCATCTTCAAGCAGAAGCGTGTTCTTGAATTGCTAGAGAA
GAACTACAAGACGATGCAGTCTCTTCAACCTGCTCAAGATTCAGAAATGGGTGGGGAA
AAGATGTCACAAGATGATGTTGCATTGCTGGCTAATATGGCTGAAGACCTGGGCATGGA
GACTAGTGACGAGAGAGAAGATGATGAAATGGATATGGATGATGCAAACATGGTAGGA
GATGGCTGTGCAAGTTTTAAAGAAAATATTATGGGAATATTGCAGCAGGGTGATTTTGC
AGAGAAGAGAGCTTCCAAACTGAGCCAAGTTGATTTCTTGTACTTGCTGTCGCTCTTC
AACAAAGCTGGTATACATTTTTCGTGA

18 SrRNA-1

ATGGCGGGCGGCAAGATCCAGAAGAAGCGGCACGGCGGAGGCGCGGGAGGCGGCGG
CGGCCGGCTGCAGGGCGGGATCCCGTTCGAGAAGTCCAAGGGGCAGCACATCCTGCG
GAACCCGGCGCTGGTGGACTCCATCGTCGCCAAGGCCGGCCTCAAGCCCACTGACAC
CGTCCTCGAGATCGGGCCCGGCACGGGGAACCTCACCAAGCGCCTGCTCGAGGCCGG
CGTCAAGGCCGTCGTCGCCGTCGAGCTCGACCCGCGCATGGTGCTCGAGCTCAACCGC
CGTTTCCAGGGGCACCCCCTCTCCTCACGCCTCAAGGTTATCCAAGGAGATGTCCTTAA
ATGTGATCTCCCGTACTTTGATATCTGTGTGGCAAACATTCCATACCAGATTTCATCCCC
CCTTACATTCAAGCTTCTGTCACACCGTCCGATCTTTAGGTGTGCTGTGATCATGTTTCA
ACGTGAATTTGCCATGAGACTTGTAGCACAGCCTGGAGACAGTCTCTACTGCCGTCTC
TCAGTGAACGTGCAGCTCTTATCACGTGTGTCACATCTCCTGAAGGTTGGACGGAACA
ACTTCAGGCCTCCACCCAAGGTTGATTCATCAGTTGTGCGTATTGAGCCCAGGAAACC
CCTCCCTCCTGTTAGCTTCAAGGAGTGGGATGGACTTGTGAGGCTTTGTTTCAATCGAA
AGAACAAGACCTTGGGCTCCATCTTCAAGCAGAAGCGTGTTCTTGAATTGCTAGAGAA
GAACTACAAGACGATGCAGTCTCTTCAACTTGCTCAAGATTCAGAAATGGGTGGGGAA
AAGATGTCACAAGATGATGTTGCATTGCTGGCTAATATGGCTGAAGACCTGGGCATGGA
GACTAGTGACGAGAGAGAAGATGATGAAATGGATATGGATGATGCAAACATGGTAGGA
GATGGCTGTGCAAGTTTTAAAGAAAATATTATGGGAATATTGCAGCAGGGTGATTTTGC
AGAGAAGAGAGCTTCCAAACTGAGCCAAGTTGATTTCTTGTACTTGCTGTCGCTCTTC
AACAAAGCTGGTATACATTTTTCGTGA

ACTIN-1

ATGGCGCAGCTGCTGGAGACGTACGCGTGCTCGCCGGCGACGGAGCGGGGCCGGGGG
ATTCTCCTCGCGGGTGACCCCAAGACGGACACGATCGCCTACTGCACCGGCCGCAGCG
TCATCATCCGCCGCCTCGATGCGCCACTTGACGCCTGGGTCTACCCCGACCACGCCTAC

CCCACTACCGTCGCCCGCTTCTCCCCCAACGGCGAGTGGGTCGCGTCCGCCGACGCTT
CCGGCTGCGTCCGCGTCTGGGGCCGCTACGGCGACCGTGCCCTTAAGGCCGAGTTCCG
CCCCCTCTCGGGCCGCGTCGACGACCTCCGGTGGTCGCCCGATGGCCTTCGCATCGTC
GTCTCTGGGGATGGAAAGGGAAAGTCCTTCGTCCGCGCATTCATGTGGGACTCTGGTA
GCACTGTTGGTGAGTTTGACGGGCACTCAAAGAGGGTTTTGAGCTGCGACTTTAAGCC
AACACGACCATTTCGGATTGTGACATGTGGTGAAGATTTTCTAGCAAACTTCTACGAGG
GACCACCATTTAAATTCAAGCATTCCATAAGGGATCACTCAAACTTTGTTAACTGTATCC
GATATTCTCCTGATGGAAGTAAGTTTATCAGTGTGAGTTCAGATAAGAAGGGTTTAATAT
ATGATGGCAAAACTGGGGAAAAGATTGGAGAGCTGTCCAGTGAAGGCAGTCACACAG
GGAGCATATATGCTGTTAGCTGGAGTCCTGACAGTAAACAAGTTCTAACGGTTTCTGCT
GATAAAACTGCGAAAGTATGGGATATCATGGAGGATGCAAGTGGGAAACTGAACAGAA
CTTTGGTTTGTCCTGGTACAGGTGGTGTAGATGACATGCTTGTGGGTTGCCTCTGGCAG
AATGATTATCTTGTGACAGTCTCTCTTGGTGGGACATTTAATCTTTTCTCCGCAAGCAAT
CCACACAAAGAACCAGTAACATTTGCAGGACATTTGAAGACTGTCAGTGCTTTGGCCT
TTTTCCCTCAAAGTAACCCAAGAACTATACTGTCTACAAGCTATGACGGTGTCATCATA
AGATGGATAGAGGGTGTTGGATATGGTGGCAGATTGATGCGTAAGAACAACACCCAGA
TCAAATGCTTTGCTGCTGCAGAAGAGGAGCTGGTCACCTCAGGGTATGATAACAAGCA
GGTCTTCATTATTCCTCTTAATGGAGATCAGTGTGGAGATGCTGAGTCAGTTGATGTAG
GAGGTCAGCCAAATGCTTTGAACCTTGCAGTTCAACGACCTGAATTTGCACTGATTAC
AACAGATTCTGGGATCATATTGCTGCACAAGTCAAAAGTCACTTCCACAACCAAAGTT
AATTATACTATCACTTCATCTGCGGTTTCCCCTGATGGCACTGAAGCCATTGTGGGTGCG
CAAGATGGGAAGCTGCGCATCTATTCCATCAGTGGGGATACACTCACAGAAGAAGCGG
TACTTGAGAAGCACCGGGGTGCTATTACTTCCATACATTATTCACCAGATGTTTCCATGT
TTGCTTCTGCTGATGCCAACAGGGAAGCTGTTGTGTGGGATCGGACAACTAGAGAGGT
GAAGTTGAAGAACATGTTGTACCACACAGCTCGGATAAACTGCCTGGCTTGGTCACCA
GACAGTAGTTTGGTTGCCACAGGTTCGTTGGACACCTGTGCAATTGTATACGAAATAGA
CAAGCCAGCATCCAGTCGCATCACCATCAAGGGAGCTCATCTTGGTGGAGTCCATGGG
TTGACCTTTGCCAGCAATGATAGTCTGGTGACTGCCGGTGAGGATGCATGCATCCGTGT
CTGGAAACTGGTGCAACAGTAG

ACTIN-2

ATGGCGCAGCTGCTGGAGACGTACGCGTGCTCGCCGGCGACGGAGCGTGGCCGGGGG
ATCCTCCTCGCTGGCGACCCCAAGACGGACACGATCGCCTACTGCACCGGCCGCAGCG
TCATCATCCGCCGCCTCGATGCGCCGCTCGACGCCTGGGTCTACCCCGACCACGCCTAC
CCCACCACCGTCGCCCGCTTCTCCCCCAATGGCGAGTGGGTCGCGTCCGCCGATGCCT
CCGGCTGCGTCCGTGTCTGGGGCCGATACGGCGACCGCGCCCTCAAGGCTGAGTTCCG
CCCCCTCTCGGGCCGCGTCGACGACCTACGCTGGTCGCCCGATGGCCTCCGCATCGTC
GTCTCCGGGGATGGCAAGGGGAAGTCCTTCGTCCGCGCATTCATGTGGGACTCTGGCA
GCACTGTTGGTGAGTTTGACGGGCACTCAAAGAGGGTTTTGAGCTGTGACTTTAAGCC
AACACGACCATTTCGGATCGTGACATGTGGCGAAGATTTTCTGGCAAACTTCTACGAG
GAACCACCATTTAAATTCAAGCATTCCATAAGGGATCACTCAAACTTTGTTAACTGTAT
CCGGTATTCTCCTGATGGAAGTAAGTTTATCAGTGTGAGTTCAGATAAGAAGGGTTTAA
TATATGATGGCAAAACTGGAGAAAAGATTGGAGAGCTGTCCAGTGAAGGCAGTCACA
CAGGGAGCATATATGCTGTTAGCTGGAGTCCTGACAGTAAACAAGTTCTAACGGTTTCT
GCTGATAAAACTGCAAAAGTATGGGATATCATGGAGGACGCAAGTGGGAAACTTAACA

GAACTTTGGTTTGTCCTGGTACAGGTGGTGTTGATGACATGCTTGTGGGTTGCCTCTGG
CAGAATGATTATCTTGTGACAGTCTCTCTTGGTGGGACATTTAATCTTTTCTCCGCGAGC
AATCCAGACAAAGAACCAGTAACATTTGCAGGACATTTGAAGGCTGCTAGTGCTTTGG
CCTTTTTTACTCAAAGTAACCCAAGAACTATACTGTCTACAAGCTATGATGGTGTCATCA
TAAGATGGATACAGGGTGTTGGATATGGAGGCAGATTGATGCGTAAGAACAACACCCA
GATCAAATGCTTTGCTGCAGCAGAAGAGGAGCTGGTCACCTCAGGGTATGATAACAAG
GTCTTCAGAATTCCTCTTAATGGTGATCAGTGTGGAGATGCTGAGTCAGTTGATGTAGG
AGGTCAGCCAAATGCTTTGAACCTTGCAGTTCAACAACATGAATTTGCACTGATTACCA
CAGATTGTGGGATCATATTGCTGCACAATTCAATAGTCACTTCCACAACCAAAGTTAAT
TACACTATCACTTCATCGGCTGTTTCTCCTGATGGCACTGAAGCCGTTGTGGGTGCGCA
AGATGGGAAACTGCACATCTATTCCATTAGTGGGGATACACTTAAAGAAGAAGCGGTA
CTTGAGAAGCACCGGGGTGCTATTACTGCCATACATTATTCGCCAGATGTTTCAATGTTT
GCTTCTGCTGATGCCAATAGGGAAGCTGTTGTGTGGGATCGGGCAACGAGAGAGGTGA
AGTTGAAGAACATGTTGTACCACACAGCTCGGATTAACTGTTTGGCTTGGTCACCGGA
CAATCGTCTGGTTGCCACAGGCTCATTGGACACCTGTGCAATTGTATACGAAATAGACA
AGCCAGCATCCAGTCGCATCACCATCAAGGGAGCTCATCTTGGCGGAGTCCATGGGTT
GACCTTTGTCGACAATGATAGTCTGGTGACTGCCGCAAAACATTTGCAGGGCATACAAT
TCTCCCCAGTGAGAGGGTAA

CYP-1

ATGGGGTTCGAGAAGCAGATCCTGAACGCCGGCACCGGCCCCAAGCCCGTCAAGGGC
CAGAAGGTCACCGTCCACTGCACCGGCTACGGGAAGGGCGGTGATCTCTCTAAGAAG
TTTTGGAGTACCAAGGACCCTGGGCAGGAGCCATTCAGTTTCAATATTGGCCTGGGCT
CAGTGATCAAAGGATGGGATGAGGGAGTTATGACCATGCAAGTGGGTGAAGTTGCTCT
GTTCTTGTTGCAGTGCACCCCAGATTATGCGTATGGAGCAGGTGGGTTTCCAGCCTGGG
GAATTAAACCAAACTCAGTGCTGATATTCGAGATTGAAGTTCTTAGCGCCCAGTGA

CYP-2

ATGGCTGCGGCGAGGCCGAAGGTGTTCTTCGACATGACGGTGGGCGGCGCTCCGGCG
GGGCGGATCGTGATAGAGCTGTTCGCTGACGACGTTCCCCGCACAGCGGAGAACTTCC
GCGCGCTGTGCACCGGGGAGAAGGGCGTGGGGCGCAGCGGCAAGCCGCTGCACTAC
AAGGGCAGCACCTTCCACCGCGTGATCCCCGACTTCATGTGCCAGGGCGGCGACTTCA
CCCGCGGCAACGGCACCGGCGGCGAGTCCATCTACGGCGAGAAGTTCGCCGACGAGA
ACTTCAAGCACAAGCACACGGCCCCCGGCATCCTCTCCATGGCCAACGCCGGGCCCA
ACACCAACGGCTCCCAGTTCTTCATCTGCACCGTGCCCTGCTCATGGCTCGATGGCAA
GCACGTCGTCTTCGGCAAGGTCGTAGACGGCCTCGATGTTGTCAAGGCCATCGAGAAG
GTCGGGTCCCGCAGCGGCTCCACTTCCAAGCCAGTCGTCATCGCCGACTGCGGCCAGC
TCTCCTAG

EF-1-α-1

ATGGGTAAGGAGAAGACCCATATTAGCATCGTGGTCATTGGCCATGTCGACTCTGGCAA
GTCGACCACCACTGGCCACCTGATCTACAAGCTTGGAGGCATTGACAAGCGTGTGATC
GAGAGGTTCGAGAAGGAAGCCGCCGAGATGAACAAGAGGTCGTTCAAGTACGCGTGG
GTGCTTGACAAGCTCAAGGCTGAGCGTGAGAGAGGTATCACCATTGATATCGCCCTGT
GGAAGTTTGAGACCACCAAGTACTACTGCACGGTCATTGATGCCCCTGGACACCGAGA
CTTCATCAAGAATATGATTACCGGTACCTCCCAGGCTGACTGCGCCGTCCTCATCATTG
ACTCCACCACCGGTGGTTTTGAGGCTGGTATCTCCAAGGATGGCCAGACCCGTGAGCA

TGCTCTCCTTGCTTTCACTCTTGGAGTGAAGCAGATGATCTGCTGCTGCAACAAGATGG
ATGCCACCACTCCCAAGTACTCGAAGGCCCGCTATGATGAAATTGTGAAGGAAGTCTC
TTCCTACCTGAAGAAGGTCGGCTACAACCCGGACAAGATTCCCTTTGTTCCCATCTCTG
GTTTCGAGGGTGACAACATGATTGAGCGGTCCACCAACCTTGACTGGTACAAGGGCCC
GACCCTGCTTGAGGCTCTTGACCAGATTAATGAGCCCAAGAGGCCATCGGACAAGCCC
CTGCGTCTCCCCCTTCAGGACGTGTACAAGATTGGTGGTATTGGTACCGTGCCTGTGGG
CCGTGTTGAGACTGGTGTCCTCAAGCCTGGCATGGTTGTTACCTTCGGTCCCAGCGGC
CTGACCACTGAGGTCAAGTCTGTTGAGATGCACCACGAGGCTCTCCAGGAGGCGCTTC
CTGGAGATAATGTTGGCTTCAACGTGAAGAACGTTGCTGTGAAGGATCTCAAGCGTGG
TTTTGTTGCTTCCAACTCCAAGGATGACCCTGCCAAGGAGGCTGCCAGCTTCACCTCC
CAGGTCATCATCATGAACCACCCTGGGCAGATTGGCAACGGCTACGCCCCAGTGCTCG
ACTGTCACACCTCCCACATTGCTGTCAAGTTTTCTGAGCTGGTGACCAAGATTGACAG
ACGATCTGGCAAGGAGCTCGAGAAGGAGCCGAAGTTCCTGAAGAACGGTGATGCTGG
CATGGTGAAGATGGTTCCCACCAAGCCCATGGTTGTGGAGACCTTCTCGCAGTACCCT
CCTCTTGGTCGTTTCGCCGTCCGTGACATGAGGCAGACTGTTGCTGTTGGTGTCATTAA
GAACGTTGAGAAGAAGGACCCAACCGGTGCCAAGGTGACCAAGGCTGCTGCCAAGA
AGAAATGA

EF-1-α-2

ATGGGTAAGGAGAAGACCCACATTAACATCGTGGTCATTGGCCATGTCGACTCTGGCA
AGTCGACCACCACTGGCCACCTGATCTACAAGCTTGGAGGCATTGACAAGCGTGTGAT
CGAGAGGTTCGAGAAGGAAGCCGCCGAGATGAACAAGAGGTCGTTCAAGTACGCGTG
GGTGCTTGACAAGCTCAAGGCTGAGCGTGAGAGAGGTATCACCATTGATATCGCCTTG
TGGAAGTTCGAGACCACCAAGTACTACTGCACGGTCATTGATGCCCCTGGACACCGGG
ACTTCATCAAGAATATGATTACCGGTACCTCCCAGGCTGACTGCGCCGTCCTCATCATT
GACTCCACCACTGGTGGTTTTGAGGCTGGTATCTCCAAGGATGGCCAGACGCGGGAGC
ACGCTCTCCTTGCTTTCACACTTGGAGTGAAGCAGATGATCTGCTGCTGCAACAAGAT
GGATGCCACCACTCCCAAGTACTCGAAGGCCCGCTATGATGAAATTGTGAAGGAAGTC
TCTTCCTACCTGAAGAAGGTCGGCTACAACCCGGACAAGATTCCCTTTGTTCCCATCTC
TGGTTTCGAGGGTGACAACATGATTGAGCGGTCCACCAACCTTGACTGGTACAAGGGC
CCGACCCTGCTTGAGGCTCTTGACCAGATCAATGAGCCCAAGAGGCCATCGGACAAGC
CCCTGCGTCTCCCCCTTCAGGACGTGTACAAGATTGGTGGTATTGGTACCGTGCCTGTG
GGTCGTGTTGAGACTGGTGTCCTCAAGCCTGGTATGGTTGTTACCTTTGGTCCCAGCGG
CCTGACCACTGAGGTCAAGTCTGTTGAGATGCACCACGAGGCTCTCCAGGAGGCACTT
CCTGGTGACAATGTTGGCTTCAATGTGAAGAACGTTGCTGTGAAGGATCTCAAGCGTG
GTTTTGTTGCTTCCAACTCCAAGGATGACCCTGCCAAGGAGGCTGCCAGCTTCACCTC
CCAGGTCATCATCATGAACCACCCTGGGCAGATTGGCAACGGCTACGCCCCAGTGCTG
GACTGCCACACCTCCCACATTGCTGTCAAGTTTGCTGAGCTGGTGACCAAGATTGACA
GACGATCTGGCAAGGAGCTCGAGAAGGAGCCCAAGTTCCTGAAGAACGGTGATGCTG
GCATGGTGAAGATGGTTCCCACCAAGCCCATGGTTGTGGAGACCTTCTCTGCGTACCC
TCCTCTCGGTCGTTTTGCCGTCCGTGACATGAGGCAGACCGTTGCTGTAGGTGTCATTA
AGAACGTTGAGAAGAAGGACCCAACCGGTGCCAAGGTGACCAAGGCTGCTGCCAAG
AAGAAATGA

GAPDH-1

ATGGGCAAGATTAAGATCGGAATCAACGGTTTCGGAAGGATCGGGAGGCTCGTGGCCA

GGGTCGCCCTCCAGAGCGAGGATGTCGAGCTCGTCGCCGTCAACGACCCCTTCATCAC
CACCGACTACATGACCTACATGTTCAAGTACGACACCGTGCACGGCCAGTGGAAGCAC
AGTGACCTCAAGATCAAGGACTCCAAGACGCTCCTCTTCGGCGAGAAGCCGGTCACT
GTCTTTGGCATAAGGAACCCTGAGGAGATCCCATGGGCTGAGGCTGGTGCTGAGTATG
TCGTGGAGTCCACTGGTGTCTTCACTGACAAGGAGAAGGCTGCTGCTCACTTGAAGG
GTGGTGCCAAGAAGGTGGTCATCTCTGCCCCAAGCAAAGATGCACCGATGTTTGTGGT
TGGTGTCAATGAGGACAAGTACACCTCTGATGTTAACATTGTCTCGAATGCTAGCTGCA
CCACAAATTGCCTTGCTCCCCTTGCAAAGGTCATTCATGACAACTTTGGTATTATTGAG
GGTCTGATGACCACTGTTCATGCCATCACTGCCACCCAGAAGACTGTTGATGGGCCCT
CAAGCAAGGACTGGAGAGGTGGCAGGGCTGCCAGCTTTAACATCATTCCCAGCAGCA
CTGGTGCTGCCAAGGCTGTTGGCAAGGTTCTTCCTGATTTGAATGGCAAGCTTACGGG
CATGTCTTTCCGGGTTCCCACCGTGGATGTGTCAGTTGTTGACCTCACTGTAAGAATCG
AGAAGGCTGCCTCATACGATGCCATCAAGAACGCTATCAAGGCGGCGTCTGAGGGAAA
GCTTAAGGGGATCATGGGTTATGTTGAGGAAGATTTGGTTTCCACCGACTTCATCGGTG
ACAGCAGGTCGAGCATCTTCGACGCCAAGGCTGGAATTGCTCTTAACGACCACTTTGT
CAAGCTTGTCTCCTGGTACGACAACGAGTGGGGTTACAGACAAAGTAGATCGTTTTGC
TTCCCATGGTTTTGCCTTAATGGCCGTGCCGGAGGCTGGCCTGCTTTGTCTATGATGAG
AATAAATGTGGATGGTAATTCACCAGACTCCCGGTTCATGCTTAGCTGGGACATGAGTT
GCGTTTTGTTTTCCTATCTACTACTACCTGTAACGAAGTTGCTAATATGGGTGGACCTGA
GTTTTACCTTTTGCTTGTGA

GAPDH-2

ATGGGCAAGATTAAGATCGGAATCAACGGTGAGTCCCTACCTGAATCTACGAATTTGTG
CCTTTTCGGTTTCGGAAGGATCGGGAGGCTCGTGGCCAGGGTCGCCCTCCAGAGCGA
GGATGTCGAGCTCGTCGCCGTCAACGACCCCTTCATCACCACCGACTACATGACCTAC
ATGTTCAAGTACGACACCGTGCACGGCCAGTGGAAGCACAGTGACCTCAAGATCAAG
GACTCCAAGACGCTCCTCTTCGGCGAGAAACCAGTCACTGTCTTTGGCATCAGGAACC
CTGATGAGATCCCATGGGCTGAGGCTGGTGCTGAGTATGTCGTGGAGTCCACTGGTGT
CTTCACTGACAAGGAGAAGGCTGCTGCTCACTTGAAGGGTGGTGCCAAGAAGGTGGT
CATCTCTGCCCCAAGCAAAGATGCACCGATGTTTGTGGTTGGTGTTAATGAGGAGAAA
TACACCTCGGATGTTAACATTGTCTCGAATGCTAGCTGCACCACAAATTGCCTTGCTCC
CCTTGCTAAGGTCATTCATGACAACTTCGGTATTATTGAGGGTCTGATGACCACTGTTC
ATGCAATCACTGCCACCCAGAAGACTGTTGATGGACCCTCAAGCAAGGACTGGAGAG
GTGGCAGGGCTGCCAGTTTTAACATCATTCCCAGCAGCACTGGTGCTGCCAAGGCTGT
TGGCAAGGTTCTTCCTGATTTGAATGGCAAGCTTACAGGCATGTCTTTCCGGGTTCCCA
CTGTGGATGTGTCAGTTGTTGATCTCACTGTTAGAATCGAGAAGGCTGCCTCATACGAT
GACATCAAGAACGCTATCAAGGCGGCATCTGAGGGAAAGCTTAAGGGAATCATGGGTT
ATGTTGAGGAAGATGTGGTTTCCACCGACTTCATTGGTGACAGCAGGTCGAGTATCTTT
GACTCCAAGGCTGGAATTGCTCTTAACGAGAACTTTGTCAAGCTTGTCTCCTGGTACG
ACAACGAGTGGGGTTACAGCAACCGTGTTGTTGACCTGATCCGCCACATGGCCAAGGC
ACAGTAG

NAC-2

ATGGCTCAAACTTGCCTGCCACCTGGCTTTCGATTCCATCCAACAGATGTTGAGCTTGT
TTCCTACTACTTAAAAAGGAAGATTATGGGAAAGAAACTTCTTGTTCAAGCTATTTCAG
AGGTTGAGCTGTACAAATTTGCTCCCTGGGATCTTCCTGATAAATCCTGTCTTCGAAGC

AAAGATCTTGAATGGTTCTTCTTTTGCCCTCGTGACAAGAAATATCCTAATGGGTCCAG
GACGAATCGTGGCACACCAAATGGTTACTGGAAAACCAGTGGAAAAGATAGAACAAT
TGAGCTTAACTCTCGCATTGTTGGATCGAAGAAAACACTGATTTTTCATGAAGGCAAG
GCACCTAAAGGAAACAGGACTGATTGGGTGATGTATGAATACAAAATGGAAGATGATG
ATTTGGTTTCAGCTGGTTTCTCAAAGGATGCTTATGTGCTCTGCAAAATTTTTAAGAAA
AGTGGCCTTGGTCCAAGGATTGGGGAGCAATATGGGGCCCCATTTAATGAAAACGAAT
GGGAAAATGCAGATGCTGCAACTTCTATGTTTCCTTTGATGTCCTCTTCAGAGTTCATA
AATTCTGCTGAGGATCCACATATTCAGCATGCTGGTACTGCTAGAGCTGTTGACCAACC
ACCTGCACATCATTCATATGCTGCTTGTGCTCAGAAGAAATCATCATCTGAGCATGTTAC
TGCTACTACTTCTGTTGAGGACCTTGTTTATGTTACTGCTGATCATGCTCTCCAGGAACT
GCCTTCTCAACAGTCTGTTAAATCTGCCGGAGATGGTGTGATTTTTGTGAACAACCGTT
CATATGAGGTCAATAATGTATACAATCCTCTTGAAATTGATGGACATTTACTGGAGGAAC
TGTCTAGGTTTCTTAATGATTCTCCTCGTCGAGACAGTCCTGTTGGAGAGCAATCTGGT
CTCCATCCAATGTCAGAAGCTGAGGCTCAAGCTTTAGAAGTAAACACTTGCGATCTCTA
CAATGAATTGGCAGGGCTTGCTGGGTTAGGTGGTGTGTCAGATGTGGATCACTTTTGTG
CTAGCAGTGTGGATGTTACTGACCAGACTTTCCATCCCACGTACTCTGAACTTGCCAGC
AGTGATGATTATTTAGAGCTGAATGATCTCATCGCTCCTGGTGCAACCTTCACCTATGAA
TTTCCCGTGCCAAGCAATGATTGGCAGTATCATTTGGATCAGTCCACATACGATGGACA
CTACGATGTTGGTGATACACTATCTTCTGCTTTTGCAGTAAGTGACTCACTTCCAACAAT
GCTAGGCGTTGTTGATGATCTGCCTACTGTTGCCAATCATGGGGACTTCGGCACAGACT
TAGTTGATACCAACTATCCAGACCCTACCATGCAGTATCCATTCTCATAA

NTB-2

ATGAACAGGGTATCCAGGCGGCGGCGTCCTCGGCTCATGCCTCCATCAAACAGGATGG
CCGAGCCCTCCAAGGTGATCCACATCCGAAACGTCGGGCATGAGATCTCCGAGAGTGA
TCTGCTCCAGGTGGTGCAGCCATTCGGCACGGTCGCGAAGCTCGTTATGCTGCGTGCC
AAGAATCAGGCCCTTATCCAGATGGAGGATTTGGCTTCTGCAGTGAGCGTGCTGCAAT
ACTACACTACGATTCAACCTAGCGTAAGAGGAAGAAATGTGTACTTGCAATTTTCATCC
CACCAGGAACTGACTACTGATCAGAGCTCTCATGGACGAAATCCTGATCAGGAAGAAC
CCAACCGAATTCTCTTAGTTACCGTTCATCATATGCTCTATCCTATAACCGTCGAAGTGC
TTCATCAAGTGTTTTCTTCTTATGGATTTGTGGAGAAGATTGTCACATTTCAAAAAACA
GCTGGTTTTCAAGCCCTCATACAGTATCAGTCACGCCAAAGTGCAATACAAGCATCCGG
TGCTTTGCATGGACGGAACATATATGATGGTTGCTGCCAACTAGATGTTCAATATTCAAA
TCTCAGCGAGTTGCAAGTTCACTACAACAATGATAGATCTAGAGATTTCACAAATCCAT
CATTGCCCACAGAACAACGCCCAAGATCCTCTCAGCAAGGTTATAATGATCCTAGTGGT
CTGTTTGGTTTTCAACAGCCTGGAGCTCCATATGCGCAGATGGGCAAGGCTGCAATGAT
TGCTGCTGCATTTGGTGGAACATTGCCTCCTGGAGTGACTGGTACCAATGACCGCTGC
ACACTCATAATTAGCAATTTGAACACTGATAAAATCGATGAGGATAAGCTCTTCAATCTA
TTTTCTATGTATGGAAATATAGTGCGAATCAAGATACTCCGCAATAAGCCAGACCATGCC
CTTATCCAGATGGCTGATGGGCTTCAGGCTGAGCTTGCTGTACAGTATTTAAAGGGAGC
AATGTTACTTGGAAAGAAACTGGAAGTGAACTTCTCGAAGTACCCAACTATTACCCCG
GCTCCTGATGCACATGACTACTCAAATTCTAATCTTAACCGATTTAACAGCAATGTGGTT
AAGAACTATCGCCACTGTTGTGCTCCAACCAAGATGATCCACATTTCAGCGCTTCCACA
AGAAATATCTGAGGATGCAATCCTTACCCATGTTGGTGAGCATGGGACTATCGTCAACT
CAAGACTGTTCGAGGCGAAAGGCAAAACACAGGCCCTTGTGTTGTTTGAAAGCGAGG

AAGAGGCAACCGAGGCGCTAGTTTCTAAGCACGCAAGCAAGCTTGAGGGAGGCACCA
TTAGGATTTCATTCTCCCAGATGCAGAACATATAG

RPL-2

ATGGCCCGGATTAAGGTGCACGAGCTGCGGGGGAAGAACAAGGCGGAGCTGCAGGCA
CAGCTCAAGGACCTCAAGGCGGAGCTCTCCCTCCTCCGCGTCGCCAAGGTCACCGGC
GGTGCCCCCAACAAGCTCTCCAAGATCAAGGTGGTGCGCACCTCGATCGCGCGCGTGC
TCACGGTGATCTCGCAGAAGCAGAAGGCGGCGCTGCGGGATGCGTACAAGAAGAAGA
AGCTGCTTCCACTCGACCTCCGCCCCAAGAAGACCCGCGCCATCCGCCGGCGCCTAAC
CAAGCACCAGCTCTCTTTGAAGACCGAGAGGGAGAAGAAGCGCGAGAAGTACTTCCC
TATGAGGAAGTATGCCATCAAGGCCTAG

TEF-1

ATGGGGAAGAGAAAGTCTGCAGCTAAGCCACCTCCTAAGAAGCGGATGGACAAGCTT
GATACTGTCTTTTCCTGCCCATTCTGCAATCATGGGAGTAGTGTTGAATGCCGAATTGAT
ATGAAGAATCTGATTGGTGAAGCTTCTTGTAGAATCTGTCAGGAAAACTTCAGCACCA
CCGTGAATGCGCTGACTGAACCTATTGATATATATAGCGAATGGATTGACGAGTGTGAG
CGTGTCAACACTGTCGAAGATGATGATGGTGCATGA

TEF-2

ATGAGGGGCGGCGGCGGCGGGGTGATGGAGGAGGACGACCGGGTGGCGCACGCGCA
GATCCCGACGAGCTTCGGCCACGAGCTCCGGGCCTGCCTCCGCTGCCGCCTCGTCAAG
ACCTACGACCAGTTCAGGGAGAACGGCTGCGAGAACTGCCCGTTCCTGGAGATGGAC
AAGGAGCACGACAATGTCGTCAACTGCACCACCCCCAACTTCACCGGAATAATCTCAT
TGATGGATCCCAGTAGGAGCTGGGCTGCTCGCTGGTTGAGAATTGGTGAGTGTTCATTC
CCGGGTGCTATACACTGGCTGTATCTGAGGAGCTCCCGGAGGAGGGATTTGCCAAGAC
AACAACGTGCACTATGTACCTCCCAAGCGTGTGTGAGCAAGCCTGAGTGGAACGCAG
CGTCCCGATCCTGA

TUBLIN-1

ATGAGAGAGTGCATCTCGATCCACATCGGCCAGGCCGGTATCCAGGTCGGAAACGCGT
GCTGGGAGCTCTACTGCCTCGAGCATGGCATCCAGGCCGATGGACAGATGCCTGGTGA
CAAGACCATTGGAGGAGGTGATGATGCTTTCAACACCTTCTTCAGTGAGACTGGTGCT
GGGAAGCATGTTCCCCGTGCTGTCTTTGTTGATCTTGAGCCTACTGTGATTGATGAGGT
GCGGACTGGCACCTACCGCCAGCTCTTCCACCCTGAGCAGCTCATCAGTGGCAAGGAG
GATGCAGCCAACAACTTTGCTCGTGGTCACTACACCATTGGCAAGGAGATTGTTGATCT
GTGCCTTGACCGCATCAGGAAGCTTGCTGACAACTGCACTGGTCTCCAGGGTTTCCTT
GTGTTTAACGCTGTTGGAGGAGGAACGGGCTCTGGCCTTGGTTCTCTTCTCCTTGAGC
GCCTTTCTGTTGACTATGGCAAGAAGTCCAAGCTTGGGTTCACTATTTACCCGTCCCCT
CAGGTCTCCACCTCTGTGGTTGAGCCATACAACAGTGTCCTGTCCACCCACTCTCTCCT
TGAGCACACTGATGTGGCTGTCCTGCTTGACAACGAGGCCATCTATGACATCTGCCGCC
GCTCCCTTGACATTGAGCGCCCGACTTACACCAACCTCAACAGGCTTGTGTCTCAGGT
CATATCGTCACTGACTGCTTCCCTGAGGTTTGATGGTGCTCTGAATGTGGATGTTAATG
AGTTCCAGACCAACCTTGTGCCCTACCCAAGGATCCACTTCATGCTTTCCTCCTATGCC
CCAGTGATATCTGCTGAGAAGGCATACCATGAGCAGCTCTCTGTTGCTGAGATCACCAA
CAGCGCCTTTGAACCTTCCTCTATGATGGCCAAGTGTGACCCACGCCACGGCAAGTAC
ATGGCCTGCTGCCTCATGTACCGTGGTGATGTTGTGCCCAAGGACGTGAATGCCGCTGT
CGCCACCATCAAGACCAAGCGCACCATCCAGTTCGTCGACTGGTGCCCCACCGGCTTC

AAGTGCGGTATCAACTACCAGCCACCAAGCGTTGTCCCTGGTGGTGACCTTGCCAAGG
TCCAGAGGGCCGTGTGCATGATCTCCAATTCCACCAGTGTTGTTGAGGTCTTCTCCCGC
ATCGACCACAAGTTTGACCTCATGTACGCCAAGCGTGCCTTCGTCCACTGGAGGGAGA
GTTCTCTGAGGCCCGTGAGGATCTTGCTGCGCTGGAGAAGGACTACGAGGAGGTTGGT
GCTGAGTTCGACGATGGTGAGGATGGTGATGAGGGCGATGAGTACTAGAGCCTCCTGC
ATCCTGGTGCTTTGTCAAGGCCTGCTACTGCTATCCTGTGATCTGCCCGAGTGGCTTTAT
CTGTCTGTCTGA

TUBLIN-2

ATGAGAGAGTGCATCTCGATCCACATCGGCCAGGCCGGTATCCAGGTCGGAAACGCGT
GCTGGGAGCTCTACTGCCTCGAGCATGGCATCCAGGCCGATGGACAGATGCCTGGTGA
CAAGACCATTGGAGGAGGTGATGATGCTTTCAACACCTTCTTCAGTGAGACTGGTGCT
GGGAAGCATGTTCCCCGTGCTGTCTTTGTTGATCTTGAGCCTACTGTGATTGATGAGGT
GCGGACTGGCACCTACCGCCAGCTCTTCCACCCTGAGCAGCTCATCAGTGGCAAGGAG
GATGCAGCCAACAACTTTGCTCGTGGTCACTACACCATTGGCAAGGAGATTGTTGATCT
GTGCCTTGACCGCATCAGGAAGCTTGCTGACAACTGCACTGGTCTCCAGGGTTTCCTT
GTGTTTAACGCTGTTGGAGGAGGAACGGGCTCTGGCCTTGGTTCTCTTCTCCTTGAGC
GCCTTTCTGTTGACTATGGCAAGAAGTCCAAGCTTGGGTTCACTATTTACCCGTCCCCT
CAGGTCTCCACCTCTGTGGTTGAGCCATACAACAGTGTCCTGTCCACCCACTCTCTCCT
TGAGCACACTGATGTGGCTGTCCTGCTTGACAACGAGGCCATCTATGACATCTGCCGCC
GCTCCCTTGACATTGAGCGCCCGACTTACACCAACCTCAACAGGCTTGTGTCTCAGGT
CATATCGTCACTGACTGCTTCCCTGAGGTTTGATGGTGCTCTGAATGTGGATGTTAATG
AGTTCCAGACCAACCTTGTGCCCTACCCAAGGATCCACTTCATGCTTTCCTCCTATGCC
CCAGTGATATCTGCTGAGAAGGCATACCATGAGCAGCTCTCTGTTGCTGAGATCACCAA
CAGCGCCTTTGAACCTTCCTCTATGATGGCCAAGTGTGACCCACGCCACGGCAAGTAC
ATGGCCTGCTGCCTCATGTACCGTGGTGATGTTGTGCCCAAGGACGTGAATGCCGCTGT
CGCCACCATCAAGACCAAGCGCACCATCCAGTTCGTCGACTGGTGCCCCACCGGCTTC
AAGTGCGGTATCAACTACCAGCCACCAAGCGTTGTCCCTGGTGGTGACCTTGCCAAGG
TCCAGAGGGCCGTGTGCATGATCTCCAATTCCACCAGTGTTGTGGAGGTCTTCTCCCGC
ATCGACCACAAGTTTGACCTCATGTACGCCAAGCGTGCCTTCGTCCACTGGGAGAGTT
CTCTGAGGCCCGTGAGGATCTTGCTGCGCTGGAGAAGGACTACGAGGAGGTTGGTGC
TGAGTTCGACGATGGTGAGGATGGTGACGAGGGCGATGAGTACTAGAGCCTCCTGCAT
CCTGGTGCTTTGTCAAGGCCTGCTACTGCTATCCTGTGATCTGCCCGAGTGGCTCTATCT
GCTATCTGTCTGTCTGAACATTTGCGTTGTGATGCTTGATTTACAACCTATGTTGTTGTA
A

UB2C-1

ATGGCGTCCAAGAGGATCCTCAAGGAGTTGAAGGACCTGCAGAAGGACCCTCCCACC
TCCTGCAGCGCAGGTCCTGTGGGTGAGGACATGTTCCATTGGCAAGCCACTATCATGG
GACCTTCAGACAGCCCATTTGCTGGTGGGGTGTTCTTGGTGAACATTCATTTCCCACCA
GATTATCCTTTCAAGCCACCGAAGGTCTCCTTCCGCACCAAGGTTTTTCACCCGAATAT
CAATAGCAACGGCAGCATTTGCCTTGACATTCTCAAGGAACAGTGGAGCCCTGCTCTT
ACCATATCGAAGGTCCTCCTGTCCATCTGTTCGCTGCTCACGGATCCGAACCCTGACGA
CCCGCTGGTCCCTGAGATCGCTCACATGTACAAGACTGATAGGGCCAAGTACGAGTCC
ACTGCGCGCTCCTGGACGCAGAAGTACGCCATGGGCTAG

UB2C-2

ATGGCGTCCAAGAGGATCCTCAAGGAGTTGAAGGACCTGCAGAAGGACCCTCCCACC
TCCTGCAGCGCAGCCTACTACAGCAACAAGGAGATCTGTCAAGAGAGGACTGTCTTTT
ACCATCTGGCTCCAGCAAATTTCTCAGGTCCTGTGGGTGAGGACATGTTCCATTGGCA
AGCCACTATCATGGGACCCACGGACAGCCCATTTGCTGGTGGGGTATTCTTGGTGAAC
ATTCATTTCCCACCGGATTATCCTTTCAAGCCACCGAAGGTCTCTTTCCGCACCAAGGT
TTTTCACCCGAATATCAATAGCAACGGCAGCATTTGCCTTGACATTCTCAAGGAACAGT
GGAGCCCTGCTCTGACCATATCAAAGGTCCTCCTGTCGATCTGTTCGCTGCTCACGGAC
CCGAACCCTGACGACCCGCTGGTCCCTGAGATCGCTCACATTTACAAGACTGATAGGG
CCAAGTACGAGTCCACTGCGCGCTCCTGGACGCAGAAGTACGCCATGGGCTAG

UBC-1

ATGTCGTCGAAGCGGATCTTGAAGGAGCTCAAGGACCTGCAGAAGGATCCCCCGACC
TCCTGCAGCGCCGGCCCTGTGGGAGAAGATATGTTCCATTGGCAGGCAACAATTATGG
GTCCATCAGATAGCCCTTATGCAGGCGGCATCTTTTTGGTTACAATTCATTTTCCTCCTG
ACTATCCATTCAAACCACCTAAGGTGGCATTCAAGACAAAGGTGTTCCATCCAAACATT
AACAGCAACGGGAGCATCTGCCTTGATATCTTGAAGGAGCAATGGAGCCCTGCACTGA
CCATTTCAAAGGTGCTCCTCTCAATCTGCTCCTTGTTGACTGATCCAAATCCTGATGAC
CCATTGGTTCCGGAGATTGCTCACATGTACAAGACCGATCGGGCAAAGTACGAGTCCA
CTGCGAGGAACTGGGCCCAGAAGTATGCAATGGGCTAG

UBC-2

ATGTCAACACCTTCAAGGAAGAGACTGATGAGGGATTTCAAACGACTGATGCAGGATC
CTCCTGCTGGCATAAGTGGGGCCCCACAGGACAACAATATAATGTTGTGGAATGCTGTC
ATTTTCGGACCTGATGATACTCCTTGGGATGGAGGTACGTTCAAGCTGACACTTCAGTT
CACTGAAGATTATCCTAACAAGCCACCTACAGTGCGATTTGTTTCTCGGATGTTTCATCC
TAACACAGTTTATGCTGATGGAAGCATATGCTTAGATATCCTACAAAACCAGTGGAGCC
CGATATATGATGTAGCCGCTATACTTACATCCATCCAGTCACTCCTTTGCGATCCAAACC
CAAATTCACCTGCCAATTCTGAAGCTGCCCGCCTATTCAGTGAGAACAAGCGGGAATA
CAACCGTAAAGTGCGTGAGATTGTGGAGCAGAGCTGGACTGCGGACTGA
