# Supplementary material for: Selection and Validation of Reference Genes in Dendrocalamus brandisii for Quantitative Real-Time PCR
Source: Plants (Basel). 2024 Aug 24;13(17):2363. doi: 10.3390/plants13172363 (PMC11396877; doi:10.3390/plants13172363)
Supplement: Supplementary file 1 [file Figure.S1.Melt curve analyses of 21 reference genes..pdf › Figure.S1.Melt curve analyses of 21 reference genes..pdf]

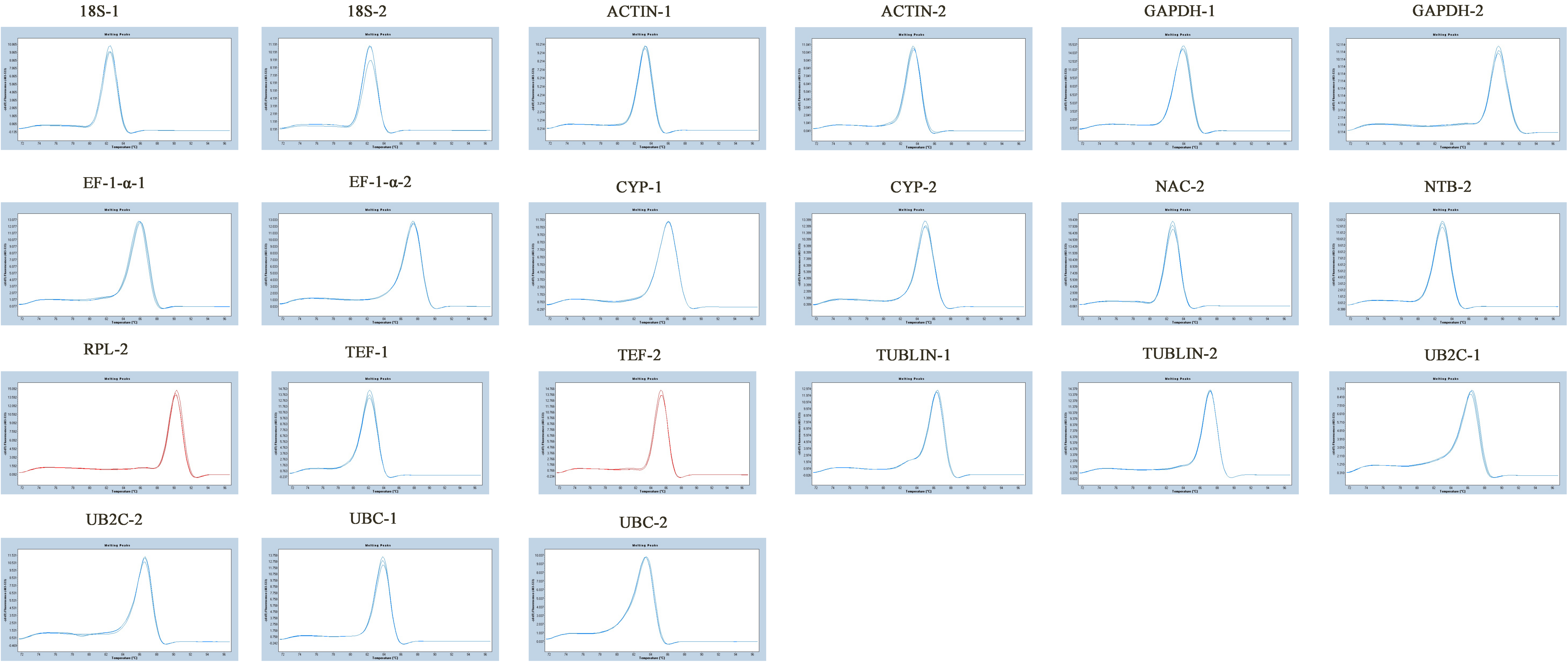
18S-1
18S-2
ACTIN-1
ACTIN-2
GAPDH-1
GAPDH-2
EF-1-α-1
EF-1-α-2
CYP-1
CYP-2
NAC-2
NTB-2
RPL-2
TEF-1
TEF-2
TUBLIN-1
TUBLIN-2
UB2C-1
UB2C-2
UBC-1
UBC-2
